# A Review of Computer-Aided Breast Cancer Diagnosis Using Sequential Mammograms

**DOI:** 10.3390/tomography8060241

**Published:** 2022-12-06

**Authors:** Kosmia Loizidou, Galateia Skouroumouni, Christos Nikolaou, Costas Pitris

**Affiliations:** 1 KIOS Research and Innovation Center of Excellence, Department of Electrical and Computer Engineering, University of Cyprus, Nicosia 2109, Cyprus; 2 Radiology Department, German Oncology Center, Limassol 4108, Cyprus; 3 Radiology Department, Limassol General Hospital, Limassol 3304, Cyprus

**Keywords:** computer-aided detection, breast cancer, mammography, sequential mammograms, review, machine learning

## Abstract

Radiologists assess the results of mammography, the key screening tool for the detection of breast cancer, to determine the presence of malignancy. They, routinely, compare recent and prior mammographic views to identify changes between the screenings. In case a new lesion appears in a mammogram, or a region is changing rapidly, it is more likely to be suspicious, compared to a lesion that remains unchanged and it is usually benign. However, visual evaluation of mammograms is challenging even for expert radiologists. For this reason, various Computer-Aided Diagnosis (CAD) algorithms are being developed to assist in the diagnosis of abnormal breast findings using mammograms. Most of the current CAD systems do so using only the most recent mammogram. This paper provides a review of the development of methods to emulate the radiological approach and perform automatic segmentation and/or classification of breast abnormalities using sequential mammogram pairs. It begins with demonstrating the importance of utilizing prior views in mammography, through the review of studies where the performance of expert and less-trained radiologists was compared. Following, image registration techniques and their application to mammography are presented. Subsequently, studies that implemented temporal analysis or subtraction of temporally sequential mammograms are summarized. Finally, a description of the open access mammography datasets is provided. This comprehensive review can serve as a thorough introduction to the use of prior information in breast cancer CAD systems but also provides indicative directions to guide future applications.

## 1. Introduction

Cancer, even after decades of research, remains a significant cause of morbidity and mortality worldwide. According to the World Health Organization (WHO) and the International Agency for Research on Cancer, there will be approximately 25 million new cancer cases and 13 million new cancer deaths by 2030. Breast cancer accounts for 11% of those cases (∼1 in 10 of all new cancer cases worldwide) and 24% of all female cancers. While the incidence rate of breast cancer is constantly increasing by 0.5% every year, the mortality has dropped by approximately 40% since the 1980s, due to the introduction of mammographic screening [1].

**Figure 1:**
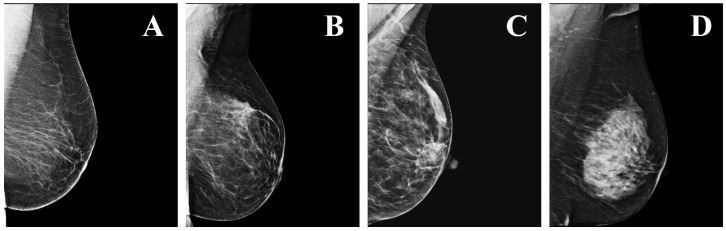
Examples of the four levels of density in mammograms, as defined by the BI-RADS: (**A**) a—almost entirely fatty; (**B**) b—scattered areas of fibroglandular density; (**C**) c—heterogeneously dense; (**D**) d—extremely dense.

Mammography, performed using low-energy X-rays, is currently the state-of-the-art method for breast cancer screening [2]. Two standard views of the breast are taken during mammography: the cranio-caudal (CC) view, taken from the top down; and the medio-lateral oblique (MLO) view, taken from the side and at an angle [3]. After the mammograms are acquired, the breast density is determined by measuring the ratio of non-dense (radiolucent) to dense (radiopaque) tissue. According to the Breast Imaging Reporting and Data System (BI-RADS) there are four density levels: (a) almost entirely fatty; (b) scattered areas of fibroglandular density; (c) heterogeneously dense, which may obscure small masses; and (d) extremely dense (Figure 1). The sensitivity of mammography decreases by increasing BI-RADS breast density. Thus, the denser the breast, the harder it is to assess the images, resulting in an increased risk of breast cancer remaining undetected [4].

Various abnormalities can be identified in a mammogram, including asymmetries between the breasts, distortion of the normal architecture, and appearance of micro-calcifications (MCs) and masses [4]. These abnormalities can be divided into two major categories depending on their severity, i.e., benign or malignant. Benign lesions are usually harmless and do not require follow-up with biopsy. However, under circumstances not well understood, they can spread to the surrounding tissues or harm nearby vital structures. Conversely, malignant abnormalities are dangerous, unstable, and require immediate follow-up, since they are associated with a very high probability of breast cancer [5]. MCs, which are small calcium deposits, are a common mammographic finding, and they typically appear as bright spots due to the high X-ray attenuation coefficient of calcium [6]. Most MCs are benign and do not require further assessment. Benign MCs are usually larger in size, with rounder and homogeneous shapes, and fewer in number. However, micro-calcification clusters (MCCs) are associated with precancerous cells or early breast cancer. Other characteristics of malignant MCCs include irregular shapes and sizes. A breast mass can be a localized swelling, protuberance, or lump, appearing as a dense region in the mammogram. Masses can be radiologically classified as benign or suspicious, depending on various parameters such as size, shape, and texture [7,8]. Benign masses are rounder, with well-defined boundaries compared to suspicious masses which have spiculated, rough and blurry boundaries [6]. When a suspicious mass is identified, its severity is confirmed by biopsy [9].

After the mammographic images are collected, two expert radiologists, along with a third if consensus is not reached, assess the images to determine whether there are any indications of malignancy. Radiologists assign one of seven assessment categories to each mammographic study: 0, needs additional imaging evaluation and/or prior mammograms for comparison; 1, negative; 2, benign; 3, probably benign; 4, suspicious for malignancy (4A, low; 4B, moderate; and 4C, high suspicion for malignancy); 5, highly suggestive of malignancy; and 6, known biopsy-proven malignancy [10]. However, since the clinicians identify signs of malignancy by visually inspecting the mammograms, misclassifications and false-positive diagnoses are inevitable. Normal breast perturbations or benign lesions, can be falsely identified as breast cancer since they can, occasionally, mimic malignant abnormalities [11]. In addition, breast masses exhibit wide variations in size, shape, and contrast and they are usually surrounded and/or enclosed by other structures, such as muscle, blood vessels, and normal breast tissue [8]. Moreover, malignant abnormalities can be missed by the radiologists due to subtle features that are difficult to perceive or due to the high density of the normal breast tissue, which reduces the visibility of the mass [12]. Studies in the literature show that the error rate in the detection of malignant masses by radiologists is approximately 10% to 30% [2].

To overcome some of the challenges in the assessment of mammography, computer-aided diagnosis (CAD) systems are being developed for the automated detection of breast abnormalities [7]. The goal of these systems is the automatic identification of subtle anomalies that might otherwise be missed by radiologists [13]. However, this task is very challenging since some abnormalities can be small (0.1–1 mm) and have various shapes and distributions, as well as low contrast, compared to normal breast tissue. Another critical challenge is the classification of breast abnormalities as benign or malignant, which often leads to a significant number of false positives (FPs) and limits the clinical applicability of CAD systems [14].

Automated diagnosis of breast cancer, based on only the most recent mammogram of a patient, usually follows three basic steps: pre-processing (using various filtering techniques); detection of the abnormality (including accurate segmentation); and classification of the detected region as normal, benign, suspicious, or malignant, depending on the study. The highest accuracy in the detection of masses with feature-based machine learning (ML) was 99.5%, achieved by Mohanty et al. who used 1500 digitized images to prove the effectiveness of their algorithm [15]. With deep learning (DL), Al-masni et al. reached 99.7% accuracy, using 600 digitized images and a convolutional neural network (CNN), but cross-validating their results only per image and not per patient [16]. Al-antari et al. achieved 97.5% accuracy in the classification of benign and malignant breast masses, using 600 digitized images, and cross-validation per patient, which is more applicable to real-world applications [17]. Despite their high accuracy, these systems are still far from clinical application. There are many reasons that can explain this paradox. These studies have been performed on different datasets, using different processing, machine learning, and validation schemes. This makes it particularly challenging to compare the studies but also to combine their results to obtain a universally applicable clinical system. Most of the open-access datasets contain outdated images, limited ground-truth annotations (i.e., bounding boxes), or they are not completely open access, requiring approval to access. Thus, various fragmented datasets with different properties and imbalanced classes are used. Furthermore, the unexplainable results of most ML models deters most clinicians from actively including such systems in their practice.

To improve their effectiveness, radiologists routinely compare the recent and prior mammograms of a patient to more effectively identify changes between screenings. Newly developed abnormalities, or regions rapidly changing between screenings, are more likely to be suspicious, compared to regions that remain unchanged and they are usually benign [18]. Prior information, when available, can provide useful insights to the clinicians, which allows them to identify possible signs of malignancy earlier and with more confidence [19]. Thus, it is reasonable to assume that the next generation of CAD systems, which can consider both the prior and recent mammograms of a patient, would lead to more accurate diagnoses.

This paper reviews the literature on the automated segmentation and/or classification of breast abnormalities from sequential mammograms, using feature-based ML and DL techniques. The first part is devoted to the importance of including prior views in the interpretation of mammography, with studies that compare the performance of radiologists with and without the use of prior mammographic images. Following, image registration techniques, which are of critical importance in the comparison of sequential mammograms, are summarized. The following section is devoted to temporal analysis of sequential mammograms for the diagnosis of breast MCs and masses. Subsequently, the implementation of detection and classification of breast abnormalities using the subtraction of temporally sequential mammograms is described. Finally, a description of the open access mammography datasets is provided. This review concludes with an overall discussion.

## 2. Review Methodology

The bibliographic literature was thoroughly searched to identify all the relevant studies. This search was limited to articles published between 2000 and 2022, written in English. Articles that included sequential data from screening methods other than mammography were excluded. Articles were also excluded if any important information regarding the algorithm’s performance was missing, making the study nonreproducible. Several review articles related to the diagnosis of breast cancer using mammograms appear in the literature [2,6,7,20,21,22,23]. However, unlike this review, none of these articles is devoted to the analysis of sequential mammograms for the detection and classification of breast cancer.

After the selection process was completed, the articles were split into two major groups, based on the approach that was used to exploit the sequential information: (a) temporal analysis, which uses both the current and prior images to extract relevant features and, then, combines them, and (b) temporal subtraction, where the prior image is subtracted from the recent one before further analysis. Subsequently, the articles in each group were further divided into two subcategories based on the breast abnormality under investigation: (a) MCs or (b) masses. Finally, each subcategory was further divided according to the classification approach: (a) not employing ML, (b) feature-based ML, or (c) DL.

Overall, there is no straightforward way to directly compare all the studies or to definitely conclude which is the most successful algorithm. The main reason is that each study is using different datasets, processing techniques, validation methods, evaluation metrics, etc. Thus, all their methodological differences must be considered when comparing results. In this review, the area under the receiver operating characteristic curve (AUC) is often used as a metric of performance to compare the various algorithms. The AUC shows the efficacy of the classification model in separating the classes; thus, the higher the AUC, the more successful the model.

## 3. Importance of Prior Views

Comparison between recent and prior mammographic views is a practice that has been employed by radiologists since the establishment of mammography as the standard screening procedure for breast cancer. During the visual inspection of images, the evolution of disease can be better assessed using sequential information, which makes any change easier to visualize. Comparisons between the images increase the effectiveness of the diagnosis and reduce the recall rates (20–50% of women recalled are found to have a malignancy [24]).

Gelig et al. evaluated the effect of the availability of prior mammograms on the performance of the radiologists during mammographic screening [25]. Three experienced radiologists assessed 150 sets of sequential mammograms twice: once without seeing the prior view (using only the most recent mammogram) and once using both the recent and prior mammographic views. The radiologists detected an average of 40 cancers with 87% specificity using only the most recent mammograms, as opposed to 37 cancers with 96% specificity when using both sequential mammograms. The increase in specificity was statistically significant, proving that the addition of the prior views reduced the recall rate. Five years later, Varela et al. also verified the importance of including prior mammograms for the classification of benign and malignant breast masses [26]. In that study, five senior and one resident radiologist evaluated 198 sequential mammograms. The mammograms were evaluated twice: once without and once with the prior images. The use of prior views increased the classification performance from 0.76 to 0.8 AUC, which was statistically significant.

**Figure 2:**
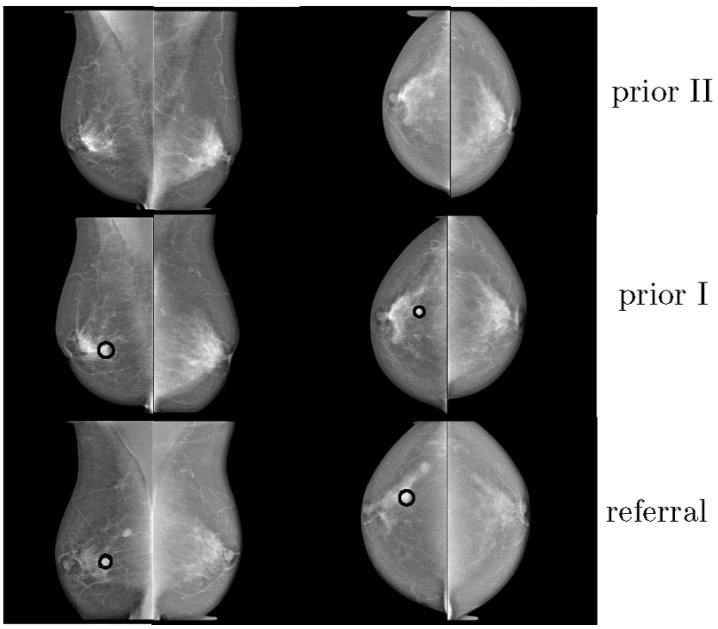
Example of three consecutive mammograms of the same woman. The last mammogram was from the time of referral. The other mammograms were obtained at previous screening rounds (reprint with permission from [24]).

Hadjiiski et al. compared the performance of eight accredited radiologists and two breast imaging fellows, with and without the use of a, so-called, interval change CAD system [27]. The software used information from prior and recent mammograms to estimate a malignancy rating. A total of 90 pairs of sequential mammograms were gathered, with 47 malignant and 43 benign masses. The introduction of the interval change analysis CAD algorithm increased the AUC from 0.83 to 0.87, proving that the analysis of prior mammograms could significantly improve the performance of the radiological assessment. Timp et al. compared the effect of a single independent reading, with a CAD system with independent double readings, for the diagnosis of breast abnormalities on 198 cases of sequential mammograms (Figure 2) [24]. Six radiologists participated in the study and three reading scenarios were considered: single reading, single reading with CAD, and independent double readings. The CAD algorithms, which included temporal information, statistically improved the diagnostic performance (0.83 vs. 0.81 AUC).

## 4. Registration

For the development of algorithms that can effectively compare sequential mammogram pairs, accurate matching between the prior and recent images, with image registration, is of critical importance. Image registration can be defined as the process of aligning two images, where one image is the reference and remains fixed and the other is the registered or moving image. The main objective is to find the optimal transformation that aligns the points of interest in the moving image to better match the fixed image. However, registration cannot be easily applied to mammograms due to the significant variations of the breast tissue between screenings, variations in breast compression, and operating factors at the time of imaging [28]. Several algorithms have been developed to address the challenges of image registration, with some approaches specifically formulated for medical images [29,30,31] and mammograms [32].

Overall, registration algorithms can be divided into “global” or “local” based on the extent of the image information used. An algorithm is classified as global if all the pixels presented in an image are used. Rigid and Affine transformations (translation, rotation, shearing) are considered global registration techniques, whereas all pixels undergo the same transformation [29]. On the other hand, an algorithm is classified as local if only some of the pixels included in a region of interest (ROI) are used at a time. Local methods, also known as deformable methods, operate on local similarities and positions and include B-spline free-form deformations [33], polyrigid transformation [34] and the Demons algorithm [35]. Registration techniques also vary with regard to the features used. Techniques based on pixel intensity are called “intensity-based”, whereas the geometrical structures of the images are known as “feature-based”. Usually, intensity-based methods are global, and feature-based methods are local. Although these methods are often applied independently, combining two or more approaches can improve the performance in terms of accuracy and robustness [31]. The combination of global and local registration algorithms, for example, can recover the main (global) scale differences but will also account for the localized nonlinear deformations (local) [32].

**Figure 3:**
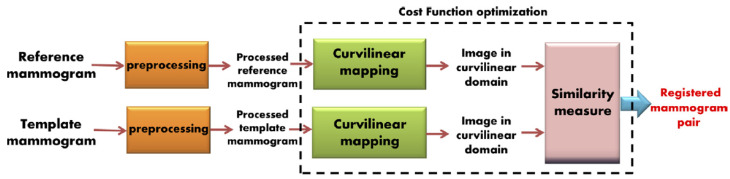
Proposed mammogram registration framework using optimized curvilinear coordinates (reprint with permission from [45]).

Various image registration techniques have been specifically applied to mammograms (Table 1). van Engeland et al. compared four different methodologies for the registration of temporally sequential mammograms. Overall, the use of mutual information provided the best performance for global mammogram registration [36]. Vujovic and Brzakovic and Marti et al. developed local registration algorithms that identified and used control points or common structures between prior and recent images, in order to establish a correspondence between those points [37,38]. Sanjay-Gopal et al., Hadjiiski et al., and Filev et al. designed computerized methods for interval change analysis, using a regional registration technique to identify corresponding lesions on temporal pairs of mammograms [19,39,40]. In a relatively recent study, Ma et al. introduced a method that incorporates fuzzy sets, based on spatial relationships, along with graph matching [41]. Hybrid registration techniques for mammogram matching have also been proposed by Wirth et al., Timp and Karssemeijer, and Li et al. [42,43,44]. A temporal mammogram registration methodology, based on the curvilinear coordinates, was proposed by Abdel-Nasser et al. (Figure 3). This method combined global and local deformations in the breast area in order to improve the registration performance [45]. Recently, Sharma et al. proposed a technique for the segmentation of breast regions using a combination of data-driven clustering and deformable image registration. This approach combines traditional segmentation approaches with ML techniques and clustering, for improved registration results [46]. Furthermore, Mendel et al. exploited B-splines and multi-resolution registration to evaluate architecture changes for cancer risk assessment [47].

## 5. Temporal Analysis

The first attempt to exploit sequential mammographic images was the application of temporal analysis. In temporal analysis, the breast abnormality is localized in the most recent mammographic view and, using image registration, the location corresponding to the abnormality is also identified in the prior mammogram. Features are extracted from both, and a new feature vector is created by subtracting the features of the prior image from those of the recent image. Several studies in the literature have assessed the effectiveness of temporal analysis for the detection and classification of breast masses and MCs.

### 5.1. Detection of Breast Masses

The algorithms for the detection of breast masses using temporal analysis can be divided into three broad categories based on the detection approach: (a) without ML, (b) with feature-based ML, and (c) with DL. The detection of breast masses using temporal analysis is indeed possible without using ML, as demonstrated by Ma et al., and Shanmugavadivu et al. [18,49]. With the addition of temporal analysis, the accuracy of the detection of masses increased, illustrating the importance of adding temporal information. The true detection rate was 80%, with 1.02 false detections per image when using just the most recent mammogram, as opposed to 0.96 false detections per image with temporal analysis [18].

Using traditional feature-based ML techniques, Zheng et al. evaluated different CAD systems, optimized for the diagnosis of breast masses, with the addition of information from prior mammograms. They observed that the performance of those systems improved (0.89 vs. 0.65 AUC) [50]. Similarly, Ma et al. incorporated a temporal registration algorithm, which verified the effectiveness of temporal analysis (0.9 vs. 0.88 AUC) [51]. Timp and Karssemeijer designed an algorithm that detected interval changes between sequential mammograms but achieved only a marginal improvement with the addition of temporal features (0.72 vs 0.71 AUC) [43]. The state-of-the-art algorithms for the detection of breast masses using temporal analysis with feature-based ML are provided in Table 2.

**Figure 4:**
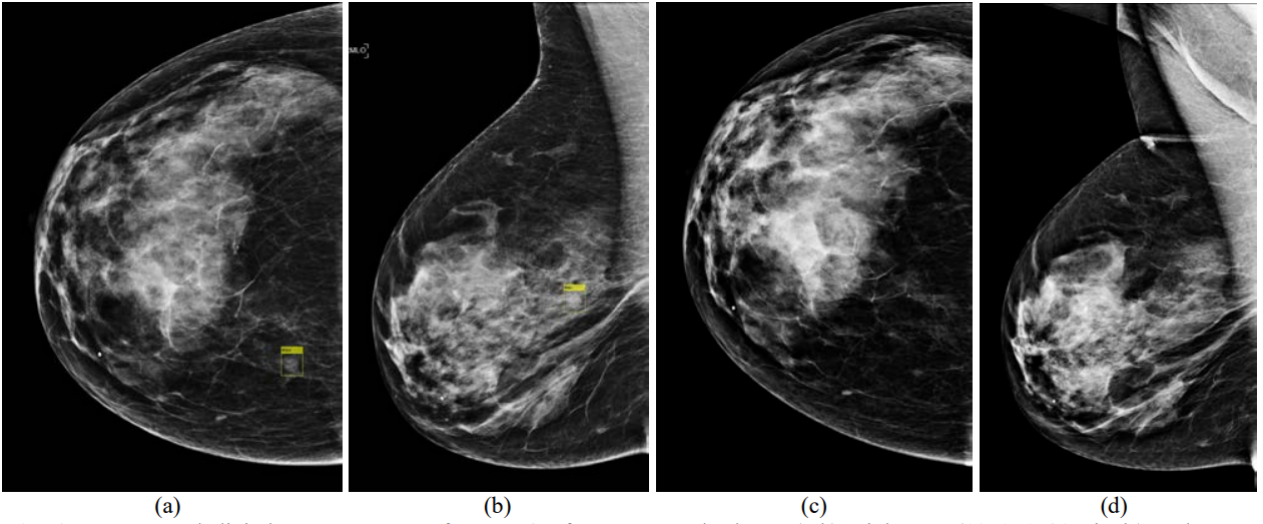
Digital mammogram pairs. (**a**,**b**) Right CC (1051 × 1521 pixels) and MLO (1069 × 1746 pixels) views of the recent mammogram with a mass present and marked by a yellow rectangle. (**c**,**d**) Right CC and MLO from a prior exam 1 year earlier (not aligned yet), which was normal (reprint with permission from [52]).

Deep learning has attracted significant attention since 2018. Kooi and Karssemeijer and Zheng et al. proposed DL algorithms for the detection of breast masses using temporal analysis (Figure 4) [52,53]. Both studies exploited deep CNNs. Their results proved that temporal analysis can improve the detection of breast masses (0.88 vs. 0.87 AUC [53]). The state-of-the-art techniques for the detection of masses in mammograms using temporal analysis with DL are provided in Table 3.

### 5.2. Classification of Breast Masses

**Figure 5:**
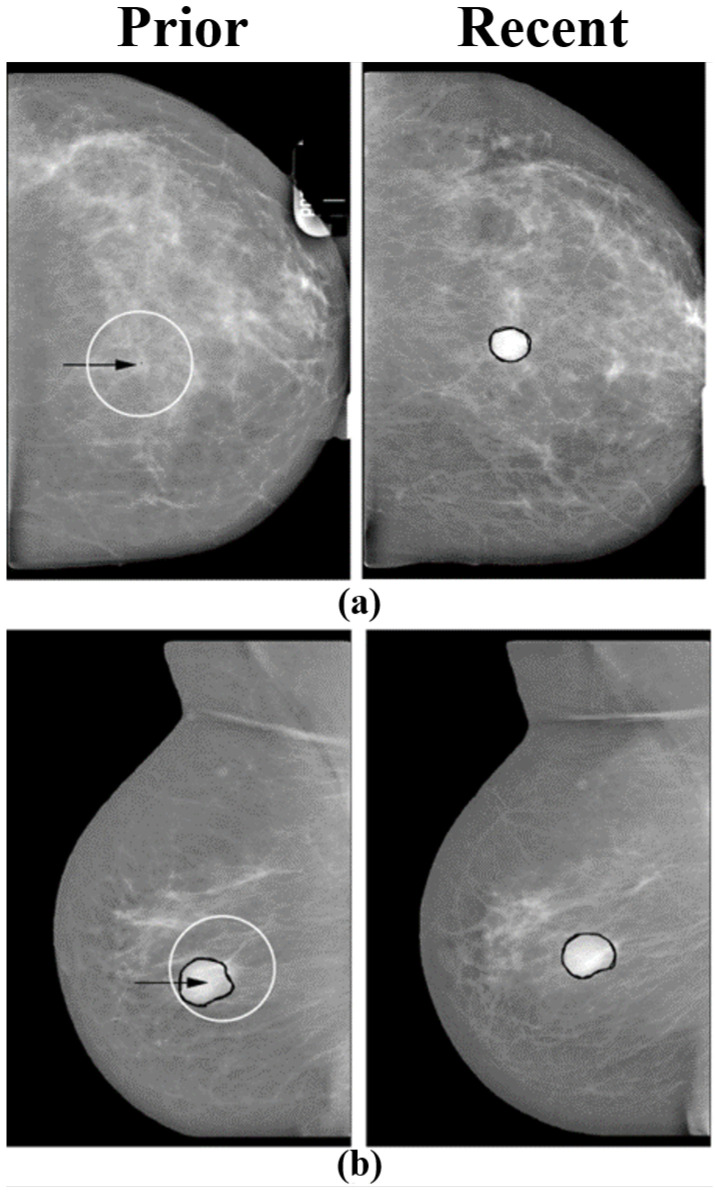
Pairs of temporally sequential mammograms. Right and left images correspond to current and prior views respectively. In each prior view, the arrow indicates the location selected by the regional registration program. (**a**) A case of a malignant mass that was not visible in the prior view. The registration program selected the most probable location in the prior view. (**b**) A case of a benign mass that was similar in the prior and current views. The registration program selected a region in the prior view that was similar to the current region (reprint with permission from [54]).

After the detection of a breast abnormality, classification usually ensues to mark the detected lesion as benign or malignant. Unfortunately, few studies have been dedicated to the classification of breast masses using sequential mammograms. One of the first groups, to use feature-based ML and temporal analysis for the classification of breast masses as benign or malignant, was Hadjiiski et al. [19]. The performance of the classification significantly improved with the addition of temporal analysis (0.88 vs. 0.82 AUC). Timp et al. and Bozek et al. also assessed the use of temporal comparison of breast abnormalities using feature-based classification [54,55]. Overall, the AUC increased significantly in both studies (0.77 vs. 0.74 [54] and 0.90 vs. 0.77 [55]) with the addition of temporal features (Figure 5). The state-of-the-art techniques for the classification of masses in mammograms using temporal analysis with feature-based ML are provided in Table 4.

### 5.3. Detection of Micro-Calcifications (MCs)

**Figure 6:**
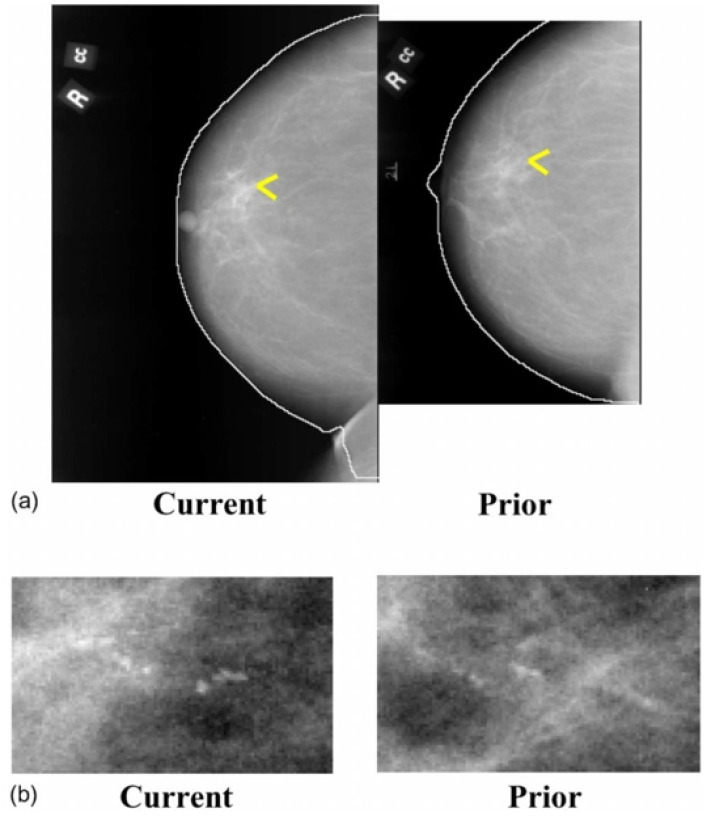
Temporally sequential mammograms containing MC clusters. (**a**) Current and prior mammograms with automatically detected breast boundaries, (**b**) A close-up of the current and prior views of the MC cluster (reprint with permission from [40]).

Several algorithms have been proposed for the detection of MCs using the most recent mammogram of a patient [21,46]. However, only Filev et al. exploited temporal analysis for this task (Figure 6) [40]. For that study, a new dataset was collected consisting of 261 pairs of digitized mammograms. First, a regional registration technique was applied to identify the area in the prior mammogram that may have corresponded to the abnormality identified in the recent mammogram. Subsequently, classification using linear discriminant analysis (LDA) and leave-one-out validation per patient, resulted in 91.2% accuracy and 0.4 FP detections per image.

### 5.4. Classification of Micro-Calcifications (MCs)

**Figure 7:**
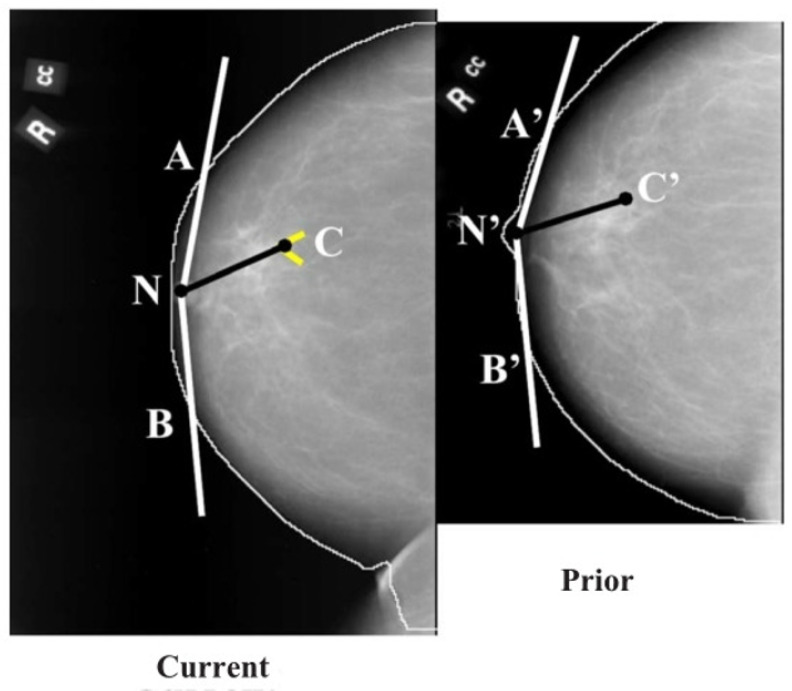
Initial estimation of the cluster centroid position in the prior mammogram based on the nipple-cluster distance and the angle between the nipple-cluster axis and breast periphery in the current mammogram (reprint with permission from [40]).

Classification of breast MCs, using the most recent mammographic view, still remains an active research topic. However, only a few studies took advantage of temporal analysis for this task. Hadjiiski et al. developed an interval change analysis algorithm for the classification of mammographic MCCs as benign or malignant [56]. Sixty-five digitized mammogram pairs were collected, and various features were extracted from each MCC. For the classification, LDA was applied with leave-one-out validation per patient. The performance significantly increased with the addition of temporal analysis (0.87 vs. 0.81 AUC). Filev et al. also showed that the performance of MC classification improved (0.81 vs. 0.72 AUC) with temporal analysis [40]. They collected 261 pairs of digitized mammograms and used regional registration, LDA, and leave-one-out validation per patient (Figure 7). The state-of-the-art techniques for the detection and classification of MCs in mammograms using temporal analysis with feature-based ML are provided in Table 5.

Despite the initial promising results, temporal analysis offers no benefit when the abnormality is new, with no traces in the prior screening examination. In addition, when small, barely discernible changes occur between screenings, they may not be apparent in the temporal feature vector and could be rejected as FPs.

## 6. Temporal Subtraction

To address some of the limitations of temporal analysis, temporal subtraction was developed by Loizidou et al. for the detection and classification of breast abnormalities [57]. The key difference between temporal analysis and temporal subtraction, is that the later exploits the entire prior image by subtracting its registered version from the entire recent image. Direct subtraction of the mammograms effectively removes the regions that have remained unchanged between screenings and enhances the contrast of new changes.

**Figure 8:**
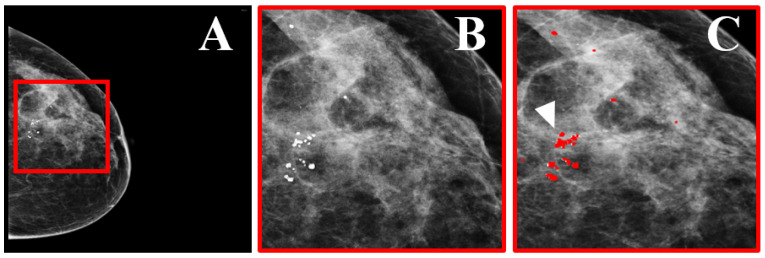
Dataset example. (**A**) Mammographic view of a woman with benign and suspicious MCs. (**B**) Zoomed region marked by the red square in (**A**), showing the MCs. (**C**) The region in (**B**) with precise marking of the MC locations (indicated by the white arrow), as annotated by two expert radiologists (reprint with permission from [58]).

Temporal subtraction was first applied for the detection and classification of breast MCs [58]. For that purpose, 100 pairs of digital mammograms were collected with precise annotation of each individual MC (benign and suspicious), as assessed by two expert radiologists (Figure 8). That dataset is now available online with open access [59]. Pre-processing, registration, subtraction, and segmentation effectively detected all ROIs that could be MCs. Machine learning was then used to reject falsely detected regions using several shape, texture, and intensity features extracted from all the ROIs. Subsequently, the correctly detected MCs were classified as BI-RADS benign or suspicious, using leave-one-patient-out cross-validation. The classification performance increased by approximately 7% in terms of accuracy (90.3% vs. 82.7%) when using temporal subtraction, as compared to using temporal analysis on the same dataset (Table 6).

**Figure 9:**
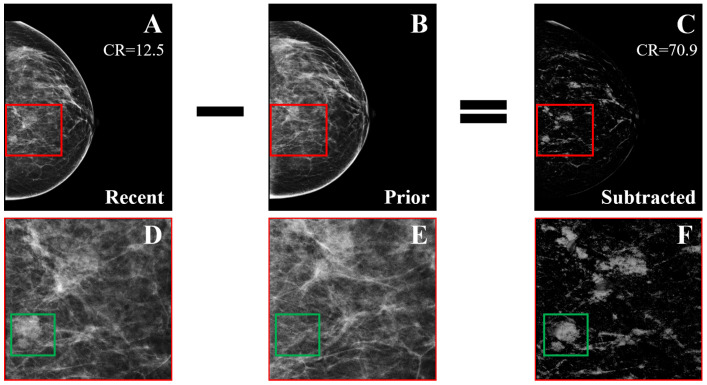
Example of temporal subtraction (BI-RADS breast density category c) with a malignant mass. (**A**) Most recent mammogram. (**B**) Prior mammogram. (**C**) The result of subtracting the registered version of (**B**) from (**A**). (**D**–**F**) Zoomed regions marked by the red squares in (**A**–**C**), respectively. The green squares enclose a new malignant mass that was not subtracted (reprint with permission from [62]).

Temporal subtraction was also applied to the detection and classification of breast masses. A new dataset was collected by Loizidou et al., consisting of 80 pairs of digital temporally sequential mammograms [60]. This dataset is also available online with open access [61]. The algorithm consists of three steps: (a) detection of the masses, which includes pre-processing, image registration, subtraction, and segmentation (Figure 9); (b) FP elimination, where falsely detected ROIs are rejected using feature extraction and ML; and (c) classification, where the detected breast masses are classified as benign or suspicious. The classifiers were trained using leave-one-patient-out cross-validation, per patient. The classification performance reached 98% accuracy when using temporal subtraction, as opposed to 92.7% when using temporal analysis on the same dataset (Table 7).

## 7. Open-Access Mammography Datasets

Various open-access mammography datasets are available online, enabling the development of reproducible algorithms and promoting the repeatability of results. However, the most commonly used datasets are relatively outdated, with scanned film mammograms, and limited ground-truth annotations or biopsy confirmations. In addition, some are not completely open access, requiring approval to use. Each dataset has its own advantages and limitations, and the choice depends solely on the needs of each particular study. Table 8 summarizes the most commonly used open-access mammography datasets, as well as their basic characteristics, such as the number of cases, the resolution (bits/pixel), the annotations available, and more. However, only two of those datasets provide sequential mammograms [59,61].

## 8. Discussion

This review summarizes the recent advances in the automated detection and/or classification of breast abnormalities using temporally sequential mammograms. Unfortunately, comparing all the existing techniques is very challenging. Although the main steps of these algorithms are similar, there are several possible approaches to implement each step and, further, to analyze the images. Another important parameter that makes the comparison between the studies difficult, is the datasets used. Open-access mammographic databases do not include sequential mammograms; thus, each research group has independently resorted to collecting sequential data. Unfortunately, only two datasets are available with open access [59,61]. Furthermore, even if the same dataset and classifier are exploited, other parameters can significantly vary. One such example is the validation method, which can significantly affect the outcome. The majority of the studies used k-fold cross-validation, with the number of k varying depending on the number of subjects. However, the construction of the training and test sets is crucial. Introducing information from the same patient in both sets, by performing cross-validation per image, or per ROI, instead of per patient, artificially increases the performance of the algorithm. All the ROIs or images of the same patient should be included either in the training or in the validation set, to avoid such bias. Unfortunately, that practise is not always adhered to, resulting in approaches that fail in real-world applications.

Despite its limitations, temporal analysis, clearly, offers an advantage in the detection and/or classification of breast masses and MCs, with a significant increase in the performance (0.77 vs. 0.90 AUC for the classification of masses [55] or 0.87 vs. 0.81 AUC for the classification of MCs [56]). However, temporal analysis offers no benefit when a newly developed abnormality appears, with few traces in the prior mammogram. To address some of the limitations, subtraction of temporally sequential mammograms exploits the whole prior screening, by subtracting the registered version of the prior images from recent ones. With direct subtraction of the mammogram pairs, ROIs that remained unchanged between screenings are effectively removed, which improves the detection and classification performance (90.3% accuracy and 0.87 AUC for the classification of MCs [58] or 98% accuracy and 0.98 AUC for the classification of masses [60]).

## 9. Conclusions

This review summarized the current trends in the analysis of sequential mammograms, exploring various concepts and methodologies. The incorporation of information from prior mammograms shows great promise for the detection of breast abnormalities. However, the lack of large-population studies limits the degree to which these results can be generalized. Furthermore, as with other CAD systems, slow clinical acceptance will probably, initially, limit temporal subtraction to a second reader role. Although, there is still a long way ahead for the translation of sequential mammogram analysis to clinical practice, the initial results, presented in this review, should encourage future research.

## Figures and Tables

**Table 1:** The state-of-the-art techniques for the registration of mammograms.

Registration Category	Reference	Details
**Global**	van Engeland et al. (2003) [36]	joint probability intensity distribution
**Local**	Vujovic and Brzakovic (1997) [37] Sanjay-Gopal et al. (1999) [39] Marti et al. (2001) [38] Hadjiiski et al. (2001) [19] Filev et al. (2008) [40] Ma et al. (2015) [41]	modified monotony operator and accumulator matrix associated information between regions correspondence between linear structures affine transformation and nonlinear optimization corresponding local search spatial relationships and graph matching
**Hybrid**	Wirth et al. (2002) [42] Timp and Karssemeijer (2006) [43] Abdel-Nasser et al. (2016) [45] Li et al. (2018) [44] Sharma et al. (2019) [48] Mendel et al. (2019) [47]	similarity and point-based spatial transformation center of mass alignment and feature space mapping curvilinear coordinates; global/local deformations global coarse and local fine registration data-driven clustering and deformable registration B-splines and multi-resolution registration

**Table 2:** Comparison of algorithms for the detection of masses in sequential mammograms using temporal analysis and feature-based machine learning.

Reference	Database	Type of Images	Dataset	Classifier	Validation Method	Result: ACC (%)	Result: SEN/SPEC (%)	Result: AUC	Result: Other
Zheng et al. (2003) [50]	Restricted	Digitized	134 pairs	ANN	75–25% (per image)	-	-	0.89 temporal 0.68 single	-
Timp and Karssemeijer (2006) [43]	Restricted	Digitized	2873 pairs	ANN	10-fold CV (per patient)	-	-	0.72 temporal 0.71 single	-
Ma et al. (2015) [51]	Restricted	Digitized	95 pairs	SMV LDA	5-fold CV (per ???)	-	-	0.9 temporal 0.88 single	-

**Table 3:** Comparison of algorithms for the detection of masses in sequential mammograms using temporal analysis and deep learning.

Reference	Database	Type of Images	Dataset	Data Augmentation	Classifier	Validation Method	Result: ACC (%)	Result: SEN/SPEC (%)	Result: AUC	Result: Other
Kooi and Karssemeijer (2017) [53]	Restricted	Digital	18366 pairs	YES	CNN (shallow gradient boosted tree)	16-fold CV (per patient)	-	-	0.88 temporal 0.87 single	-
Zheng et al. (2018) [52]	Restricted	Digital	96 pairs	NO	CNN (VGG-19)	10 × 75–25% (per image)	-	92.8/99.1	-	0.004 FPi

**Table 4:** Comparison of algorithms for the classification of masses in sequential mammograms using temporal analysis and feature-based machine learning.

Reference	Database	Type of Images	Dataset	Classifier	Validation Method	Result: ACC (%)	Result: SEN/SPEC (%)	Result: AUC	Result: Other
Hadjiiski et al. (2001) [19]	Restricted	Digitized	140 pairs	LDA	leave-one-out (per patient)	-	-	0.88 temporal 0.82 single	-
Timp et al. (2007) [54]	Restricted	Digitized	465 pairs	SVM	20-fold CV (per ???)	-	-	0.77 temporal 0.74 single	-
Bozek et al. (2014) [55]	Restricted	Digital	60 pairs	LDA	leave-one-out CV (per ???)	-	-	0.90 temporal 0.77 single	-

**Table 5:** Comparison of algorithms for the diagnosis of MCs in sequential mammograms using temporal analysis and feature-based machine learning.

Reference	Database	Type of Images	Dataset	Classifier	Validation Method	Result: ACC (%)	Result: SEN/SPEC (%)	Result: AUC	Result: Other
**Detection**									
Filev et al. (2008) [40]	Restricted	Digitized	261 pairs	LDA	leave-one-out (per patient)	91.2	-	-	0.72 FPs per image
**Classification**									
Hadjiiski et al. (2002) [56]	Restricted	Digitized	65 pairs	LDA	leave-one-out (per patient)	-	-	0.87 temporal 0.81 single	-
Filev et al. (2008) [40]	Restricted	Digitized	261 pairs	LDA	leave-one-out (per patient)	-	-	0.81 temporal 0.72 single	-

**Table 6:** Comparison of algorithms for the diagnosis of MCs in sequential mammograms using temporal subtraction and feature-based machine learning.

Reference	Database	Type of Images	Dataset	Classifier	Validation Method	Result: ACC (%)	Result: SEN/SPEC (%)	Result: AUC	Result: Other
**Detection**									
Loizidou et al. (2021) [58]	Open access	Digital	100 pairs	Voting	leave-one-out (per patient)	94.1	81.4/95.5	0.88	-
**Classification**									
Loizidou et al. (2021) [58]	Open access	Digital	100 pairs	ANN	leave-one-out (per patient)	90.3	81.6/92.2	0.87	-

**Table 7:** Comparison of algorithms for the diagnosis of masses in sequential mammograms using temporal subtraction and feature-based machine learning.

Reference	Database	Type of Images	Dataset	Classifier	Validation Method	Result: ACC (%)	Result: SEN/SPEC (%)	Result: AUC	Result: Other
**Detection**									
Loizidou et al. (2022) [60]	Open access	Digital	80 pairs	ANN	leave-one-out (per patient)	99.9	96.6/99.9	0.98	-
**Classification**									
Loizidou et al. (2022) [60]	Open access	Digital	80 pairs	ANN	leave-one-out (per patient)	98	99/96.1	0.98	-

**Table 8:** Comparison of the most commonly used open-access mammography datasets.

Name	Origin	Year	File Access	Number of Cases	Number of Images	Resolution (bits/pixel)	Image Mode	Type of Abnormality	Image Categories	Annotation
**DDSM** [63]	USA	1999	Open	2620	10480	8 or 16	Digitized	ALL	Normal Benign Malignant	Contour points of the ROI
**MIAS** [64]	UK	2003	Open	161	322	8	Digitized	ALL	Normal Benign Malignant	Center and radius of a circle around ROI
**INbreast** [65]	Portugal	2011	Approval from authors	115	410	14	Digital	Masses Calcifications Distortions Asymmetries	Normal Benign Malignant	Contour points of the ROI
**BCDR-FM** [66]	Portugal	2012	Open (requires registration)	1125	3703	8	Digitized	ALL	Normal Cancer	Precise lesion locations and mass coordinates, detailed segmentation outlines
**BCDR-DM** [66]	Portugal	2012	Open (requires registration)	1042	3612	14	Digital	ALL	Normal Cancer	Precise lesion locations and mass coordinates, detailed segmentation outlines
**CBIS-DDSM** [67]	USA	2017	Open	1566	10239	8 or 16	Digitized	Mass Calcifications	Benign Malignant	ROI segmentation and bounding boxes
**DDSM-BCRP** [68]	USA	2000	Open	179	716	12	Digitized	Masses Calcifications	Benign Malignant	Contour points of the ROI
**OPTIMAM** [69]	UK	2020	Approval from authors	-	2889312	12 or 16	Digital	ALL	Normal Benign Malignant	Rectangular around the boundaries of the ROI
**SDM-MCs** [59]	Cyprus	2021	Open	100	400	12	Digital	Calcifications	Normal Benign Suspicious	Precise annotation of each micro-calcification
**SDM-Masses** [61]	Cyprus	2022	Open	80	320	12	Digital	Masses	Normal Benign Malignant	Precise annotation of each mass

## Data Availability

Not applicable.
